# ﻿An update to the taxonomy of *Serica* MacLeay, 1819 (sensu lato) from China (Coleoptera, Scarabaeidae, Sericinae, Sericini)

**DOI:** 10.3897/zookeys.1185.109154

**Published:** 2023-11-28

**Authors:** Xie Su, Wei Li, Wan-Gang Liu, Dirk Ahrens, Ying-Juan Zheng, Yang Liu

**Affiliations:** 1 Key Laboratory of Resource Biology and Biotechnology in Western China (Northwest University), Shaanxi Key Laboratory for Animal Conservation, College of Life Science, Northwest University, Taibai North Road 229, Xi’an 710069, China Northwest University Xi'an China; 2 SKLLQG, Institute of Earth and Environment, Chinese Academy of Sciences, Yanxiang Road 97, Yanta District, Xi’an 710061, China SKLLQG, Institute of Earth and Environment, Chinese Academy of Sciences Xi'an China; 3 Zoologisches Forschungsmuseum A. Koenig, Adenauerallee 127, 53113 Bonn, Germany Zoologisches Forschungsmuseum A. Koenig Bonn Germany; 4 Chinese Research Academy of Environmental Science, Beijing 100012, China Chinese Research Academy of Environmental Science Beijing China

**Keywords:** Beetles, chafers, key, new species, new records, Yunnan

## Abstract

In this paper we update the knowledge on the species of *Serica* McLeay, 1819 (sensu lato) occurring in Yunnan, Sichuan, and Shaanxi provinces, China. Three new species are described: *Sericaallonanhua* Liu, Ahrens, Li & Su, **sp. nov.**, *S.breviantennalis* Liu, Ahrens, Li & Su, **sp. nov.**, and *S.fengensis* Liu, Ahrens, Li & Su, **sp. nov.** The key to the species groups and species is updated. The habitus and male genitalia of the new species are illustrated, and a map showing their distribution is provided. New distributional data are given for four species.

## ﻿Introduction

The tribe Sericini Kirby, 1837 includes more than 200 genera and 4000 species. While the phylogenetic position of Sericini appears now to be resolved, its internal relationships still await refinement with additional sampling of taxa and genetic markers needed (Dietz et al. 2023).

The genus *Serica* was established by Macleay (1819), and the type species, by monotypy, is *Sericabrunnea* (Linnaeus, 1758) (Ahrens 1999, 2005a). It is one of the most diverse sericine genera in the Palaearctic Region, with hundreds of species in the temperate regions of Eurasia and higher mountains of Asia (Ahrens 2005a, 2006b, 2007b; Ahrens and Bezděk 2016). Under currently understood, *Serica* is polyphyletic, as shown by a series of molecular phylogenetic studies (Ahrens and Vogler 2008; Liu et al. 2015b; Eberle et al. 2017). Ahrens (1999, 2005a) established the subgenus Serica (sensu stricto) for the species most closely related to the type species, *S.brunnea* (Linnaeus, 1758). Molecular evidence suggests that many species with an elongate body shape, trilamellate antennal club, and narrower, longer legs have been placed incorrectly in *Serica* by many authors due to convergence of these characters (Ahrens 1999, 2005a). As a consequence of the often occurring strong homoplasy, many diagnostic characteristics of Sericini have been proven to be misleading for higher classification, including the number of antennomeres which traditionally has been prominently used for the definition of genera of Sericini (Ahrens and Vogler 2008). This had led to many unresolved problems in generic classification, especially in the “*Maladera–Neoserica–Serica*” clade (Eberle et al. 2017). Therefore, many of our recent taxonomic treatments of *Serica*, *Maladera* Mulsant & Rey, 1871, and *Neoserica* Brenske, 1894 divided these genera into compact species groups or subgenera, which are likely to represent monophyletic groups, as a basis for future systematic work (e.g. Ahrens and Fabrizi 2016; Fabrizi et al. 2021). These are generally summarized under “sensu lato” in association with the respective genus name, or as subgenera. The subgenus Serica (sensu stricto) has had a vast radiation in the Asian highlands, mainly the Himalayas, eastern Tibet, and the mountains of the eastern Palaearctic (Ahrens 2007c).

This study is another contribution to a comprehensive taxonomic revision of the Sericini of China and adjacent regions. Previous works have comprised taxonomic revisions of all genera occurring in China to which also already several updates have been added (Ahrens 1996, 2000a, b, c, 2001, 2003a, b, c, d, 2005a, 2005b, 2006a, b, c, 2007a, b, c, 2009, 2011, 2021, 2023a, b; Ahrens and Pacholátko 2003, 2007; Ahrens and Fabrizi 2009a, 2009b, 2011; Liu et al. 2011, 2014a, b, c, d, e, 2015a, 2016, 2017a, 2017b, 2019; Ahrens et al. 2014a, b, c, 2021a, 2021b; Bohacz and Ahrens 2020; Fabrizi et al. 2021; Ahrens et al. 2022, 2023; Zhao and Ahrens 2023). Recently, the species of *Serica* MacLeay, 1819 (sensu lato) from China and adjacent areas were revised comprehensively (Ahrens et al. 2022). In this paper, we provide further updates on *Serica* (s.l.) species in China, which include new records and the description of three new species.

## ﻿Materials and methods

The terminology and methods used for measurements, specimen dissection, and genital preparation follow Ahrens (2004). Data from specimens examined are cited in the text with original label contents given in quotation marks; multiple labels for a single specimen are separated by a “/”. Descriptions and illustrations of new taxa are based on the holotype specimen if not otherwise stated, while the variation of specimens is given separately under “variation”. Male genitalia were glued to a small, pointed card and photographed in both lateral and dorsal views using an Olympus SZ61-TR stereomicroscope with an Mshot MD 50 digital camera. All the parameter adjustment and images acquisition were done in MShot Image Analysis System v. 1.6.1. To obtain an focused image, several partly focused images were also focus-stacked with this software. The resulting images were subsequently digitally edited with Adobe Photoshop v. 21.0.2. Distribution maps were generated using ArcGIS v. 10.3, based on the geographical coordinates from the labels.

Abbreviations used in the text for collection depositories are as follows:

**SENU**Laboratory of Systematic Entomology, Northwest University, Xi’an, Shaanxi

**IZAS**Institute of Zoology, Chinese Academy of Sciences, Beijing, China

**ZFMK**Zoologisches Forschungsinstitut und Museum A. Koenig, Bonn, Germany

## ﻿Results

### ﻿Key to males of *Serica* (sensu lato) species of continental South-East Asia

**Table:** 

1	Dorsal surface unicoloured	**2**
–	Dorsal surface, particularly elytra, multicoloured	**28**
2	Dorsal surface brown and glabrous	**3**
–	Dorsal surface darker and generally with erect setae on dorsal surface	**12**
3	Body reddish to dark brown. Antennal club at least twice as long as remaining antennomeres combined	**4**
–	Body yellowish brown. Antennal club at maximum 1.5 times as long as remaining antennomeres combined	**8**
4	Metatibia long and narrow, ratio width:length 1:4.2. Antenna with 10 antennomeres	**5**
–	Metatibia long and little wider, ratio width:length 1:3.7	**6**
5	Metatibia long and narrow, ratio width:length 1:4.2. Apex of the phallobase strongly bent in lateral view	***S.solivaga* (Brenske, 1898)**
–	Metatibia moderately long and wide, ratio width:length 1:3.2. Apex of the phallobase more straight (lateral view)	***S.mengsonana* Ahrens, Fabrizi & Liu, 2022**
6	Antenna with 9 antennomeres. Antennal club 5 times as long as remaining antennomeres combined. Basal lobe of the right paramere wide and large	***S.fashengi* Liu & Ahrens, 2014**
–	Antenna with 10 antennomeres. Antennal club 4 times as long as remaining antennomeres combined. Basal lobe of the right paramere normal or narrow	**7**
7	Right paramere narrower, without blunt dorsomedian extension, sharply pointed towards apex in lateral view. Dorsal lobe of left paramere externally reflexed	***S.falcifera* Ahrens & Fabrizi, 2009**
–	Right paramere wider, with a blunt dorsomedian extension, moderately pointed towards apex in lateral view. Dorsal lobe of left paramere internally reflexed	***S.segregata* Arrow, 1946**
8	Dorsal surface dull. Phallobase at right side with an apical lateral apophyses laterally	**9**
–	Dorsal surface shiny. Phallobase at right side without apical lateral apophyses laterally	***S.longwan* Ahrens, Fabrizi & Liu, 2022**
9	Basal lobe of left paramere extended only basomedially	**10**
–	Basal lobe of left paramere extended basomedially and distally	***S.longula* Frey, 1972**
10	Basal lobe of left paramere strongly curved and directed distally	***S.tiammushan* Ahrens, Fabrizi & Liu, 2022**
–	Basal lobe of left paramere medially directed	**11**
11	Antennal club distinctly longer than remaining antennomeres combined. Right paramere moderately dorsally angled	***S.daqiaoana* Ahrens, Fabrizi & Liu, 2022**
–	Antennal club distinctly equal to length remaining antennomeres combined. Right paramere strongly dorsally angled	***S.jiankouensis* Ahrens, Fabrizi & Liu, 2022**
12	Labroclypeus distinctly medially emarginate	**13**
–	Labroclypeus weakly medially emarginate	**15**
13	Dorsal surface shiny. Aedeagus in apical half dorsoventrally flattened	***S.excisa* (Frey, 1972)**
–	Dorsal surface dull. Aedeagus in apical half almost spherical in cross section	**14**
14	Labroclypeus deeply medially emarginate. Lateral margin of pronotum slightly concave	***S.shiduensis* Ahrens, Fabrizi & Liu, 2022**
–	Labroclypeus moderately medially emarginate. Lateral margin of pronotum straight	***S.klapperichi* Frey, 1972**
15	Body more oval, pronotum subequal, a third of elytral length	**16**
–	Body elongate, pronotum subequal, a quarter of elytral length	**17**
16	Antennal club in male long, three times as long as remaining antennomeres combined. Parameres with distinct basal lobes	***S.huangbaiyuanensis* Ahrens, Fabrizi & Liu, 2022**
–	Antennal club in male short, as long as remaining antennomeres combined. Parameres without distinct basal lobes	***S.guangnanensis* Ahrens, Fabrizi & Liu, 2022**
17	Parameres at maximum almost subequal to one-half length of phallobase	**18**
–	Parameres distinctly shorter	**19**
18	Left paramere almost perpendicular to right one; club longer than twice of remaining antennomeres combined	***S.lupina* Arrow, 1946**
–	Left paramere almost parallel to or forming an acute angle with right one; club shorter than twice the length of remaining antennomeres combined	***S.breviantennalis* Liu, Ahrens, Li & Su, sp. nov.**
19	Parameres at maximum subequal to one-third of phallobase length	**20**
–	Parameres at maximum subequal to one-quarter or less of phallobase length	**21**
20	Right paramere curved ventrally in lateral view, without preapical lateral tooth	**22**
–	Right paramere straight in lateral view, with sharp and distinct preapical lateral tooth	***S.yini* Ahrens, Fabrizi & Liu, 2022**
21	Dorsal surface of body densely setose. Left paramere nearly straight, distinctly shorter than right one	***S.baishuitaiensis* Ahrens, Fabrizi & Liu, 2022**
–	Dorsal surface of body sparsely setose. Left paramere strongly curved and bent ventrally below ventral portion of phallobase, almost as long as right paramere	***S.paralupina* Ahrens, Fabrizi & Liu, 2022**
22	Apex of right paramere sharply pointed	***S.gaoligong* Ahrens, Fabrizi & Liu, 2022**
–	Apex of right paramere rounded or bluntly truncate	**23**
23	Dorsal pilosity dense	**24**
–	Dorsal pilosity sparse	**27**
24	Left paramere weakly curved	**25**
–	Left paramere strongly curved. Right paramere weakly curved in dorsal view	***S.longipes* Ahrens, Fabrizi & Liu, 2022**
25	Parameres shorter, at maximum one-sixth as long as phallobase. Left paramere strong, obviously shorter than right one. Right paramere weakly curved in dorsal view	***S.nanhua* Ahrens, Fabrizi & Liu, 2022**
–	Parameres longer, at least one-quarter as long as phallobase. Left paramere narrower and slightly shorter than right one. Right paramere strongly curved in dorsal view	**26**
26	Parameres without basal lobes	***S.allonanhua* Liu, Ahrens, Li & Su, sp. nov.**
–	Parameres with long and distinct basal lobes	***S.fengensis* Liu, Ahrens, Li & Su, sp. nov.**
27	Right paramere strongly curved. Left paramere wide, flattened in cross section over almost two-thirds of its entire length	***S.allolongipes* Ahrens, Fabrizi & Liu, 2022**
–	Right paramere weakly curved. Left paramere narrow, sickle-shaped, and almost circular in cross section over almost its entire length	***S.paralongipes* Ahrens, Fabrizi & Liu, 2022**
28	Parameres inserted strongly asymmetric; left paramere much more basally than the right one	**29**
–	Phallobase rather symmetric; parameres inserted almost at the same level	**37**
29	Right paramere with a narrow basal lobe directed medially	**31**
–	Right paramere without a narrow basal lobe directed medially. Phallobase narrowed towards apex in dorsal view	**30**
30	Phallobase concavely dorsoapically excised	***S.dathei* Ahrens, 2005**
–	Phallobase with a process-like median extension at mesoapical margin. Antennal club 1.2 times as long as remaining antennomeres combined	***S.dissimillima* Ahrens, Fabrizi & Liu, 2022**
31	Phallobase truncate apex (dorsal view), its dorsal margin straight. Antennal club 1.5 times as long as remaining antennomeres combined	**32**
–	Phallobase concavely dorsoapically excised	**33**
32	Left paramere more than one-half length of right paramere	***S.zhenba* Ahrens, Fabrizi & Liu, 2022**
–	Left paramere less than one-half length of right paramere	***S.nanjiangana* Ahrens, 2005**
33	Right paramere very long and narrow, sharply pointed. Left paramere without any lateral tooth	***S.trapezicollis* Ahrens, 2005**
–	Right paramere moderately long and wider, rounded at apex	**34**
34	Left paramere about one-third length of right paramere	**35**
–	Left paramere more than one-half length of right paramere	**36**
35	Right paramere abruptly narrowed behind middle. Left paramere not dorsoventrally flattened	***S.jani* Ahrens, Fabrizi & Liu, 2022**
–	Right paramere evenly narrowed towards apex. Left paramere dorsoventrally flattened	***S.jaroslavi* Ahrens, Fabrizi & Liu, 2022**
36	Left paramere in distal portion with a lateral tooth	***S.albisetis* Ahrens, 2005**
–	Left paramere without lateral apical tooth, but its left paramere having its dorsomedial tooth at basal third	***S.shengtangshan* Ahrens, Fabrizi & Liu, 2022**
37	Aedeagus robust, with a long double dorsal phallobasal process which exceeds almost parameres	***S.adspersa* Frey, 1972**
–	Aedeagus elongate, without dorsal phallobasal process, but simply concavely emarginate between parameres	**38**
38	Elytral surface with moderately dense to dense setae	**39**
–	Elytral surface with sparse setae	**40**
39	Pilosity of dorsal surface double, with at least two types of setae. Elytra with a large dark spot before apex	***S.nigroguttata* group**
–	Pilosity of dorsal surface simple, with one type of setae only. Elytra without dark spot before apex. Metasternum anteriorly abruptly and strongly elevated	***S.velutina* group**
40	Parameres both subdivided into a long dorsal and ventral lobe. Metafemur, submarginal serrate line beside anterior margin present or absent	**Serica (subgenus Taiwanoserica Nomura, 1974)**
–	Parameres not subdivided into a long dorsal and ventral lobe, or only one of both subdivided into a long dorsal and ventral lobe. Metafemur, submarginal serrate line beside anterior margin always absent	**41**
41	Pronotum almost glabrous, with a few adpressed setae on posterior disc; surface evenly plane, in anterior part shiny, posteriorly dull. Frons shiny in anterior half	***S.ziqingi* Ahrens, Fabrizi & Liu, 2022**
–	Pronotum densely setose on anterior disc, surface with shallow transverse impressions, entirely dull or iridescent shiny. Frons entirely dull	**42**
42	Pronotum narrow. Body more elongate. Parameres shorter, left one half as long as right	***S.jirii* Ahrens, Fabrizi & Liu, 2022**
–	Pronotum more transverse. Body oblong and less convex. Parameres long, equal in length	***S.pangwa* Ahrens, Fabrizi & Liu, 2022**

### ﻿Species descriptions

#### ﻿ Serica breviantennalis

Taxon classificationAnimaliaColeopteraScarabaeidae

Liu, Ahrens, Li & Su sp. nov.

B6D2407A-766D-57AB-A959-F4058825301B

https://zoobank.org/09579AA9-C713-4E38-8CBD-921A41747FCF

Figs 1A–H , 3

##### Type materials.

***Holotype***: ♂ [China] “Black Tiger Mountain, Tianxin Township, Wuding County, Chuxiong, Yunnan Province, 20.V.2022, light trap, leg. Zhao Yuchen, Lu Jinbo” (SENU). ***Paratypes***: 28 ♂♂ [China] “Black Tiger Mountain, Tianxin Township, Wuding County, Chuxiong, Yunnan Province, 20.V.2022, light trap, leg. Zhao Yuchen, Lu Jinbo” (SENU, IZAS, ZFMK), 2 ♂♂ [China] “Gaojianchou village, Yao ‘an County, Chuxiong, Yunnan Province, 24.V.2022, light trap, leg.Xu Rentao” (SENU), 2 ♂♂ [China] “Xiaogupu Village, Shangdianliang Road, Wuding County, Chuxiong Autonomous Prefecture, Yunnan Province, 21.V.2022, light trap, 2092.6m, 25.6724°N, 102.2782°E, leg. Lu Jinbo” (SENU), 1 ♂ [China] “Zhangjiacun Mixing Town, Yao ‘an County, Chuxiong City, Yunnan Province, 23.V.2022, light trap, 25.4041°N, 101.1683°E” (SENU).

##### Description of the holotype.

Length 7.9 mm, elytral length 5.6 mm, width 4.1 mm. Body oblong, reddish brown; frons dark reddish brown, dull; legs yellowish brown; antenna yellow, with moderately long setae on head dorsal surface.

Labroclypeus wide, trapezoidal, with lateral margins moderately convex and convergent, producing a blunt angle with ocular canthus; anterior angles strongly convex; anterior margin distinctly emarginate medially; margins moderately reflexed; surface moderately convex, finely and densely punctate, with numerous long and erect setae; frontoclypeal suture distinct, moderately curved; ocular canthus moderately narrow (length slightly >1/3 ocular width), smooth and glabrous, with four terminal setae. Frons with dull toment, with fine, moderately dense punctures, anteriorly and laterally with a few long setae. Antenna with nine antennomeres, club with three antennomeres, 1.5 times as long as remaining antennomeres combined, distinctly reflexed. Eyes small, ratio diameter to interocular width 0.47. Mentum convexly elevated and flattened anteriorly.

***Pronotum*** moderately wide, widest at base, lateral margins moderately convex and convergent anteriorly; anterior angles acute, moderately produced, posterior angles blunt; anterior margin weakly convex, marginal line fine but complete; basal margin without marginal line; surface with coarse, dense punctures and minute setae, anteriorly and laterally bearing erect long setae. Hypomeron not carinate ventrally. Scutellum narrow, long, triangular, with fine but dense punctation and minute setae in punctures, and basally with a triangular smooth area.

***Elytra*** elongate, narrow, widest in posterior third; striae distinctly impressed, finely punctate; intervals weakly convex, finely and densely punctate, with punctures concentrated along striae; elytra glabrous, lateral intervals with a few moderately long, adpressed or erect setae; epipleural margin robust, ending at strongly rounded external apical angle of elytra; epipleura sparsely setose; apex of elytra with a very fine, membraneous rim of short microtrichomes.

***Ventral surface*** finely and densely punctate, glabrous. Metacoxae laterally with a few longer setae. Mesosternum between mesocoxae slightly wider than mesofemur. Ratio of length of metepisternum to metacoxa 1:1.52. Abdominal sternites finely and moderately densely punctate, each with a row of coarse punctures, each puncture bearing each a robust seta. Pygidium almost flat and finely, densely punctate, with moderately dense, long setae.

***Legs*** long, narrow, shiny; femora finely, sparsely punctate, with two longitudinal rows of setae. Metafemur with anterior margin acute, without adjacent serrate line; posterior ventral margin almost straight, weakly widened in apical half, ventrally and dorsally not serrate, sparsely setose. Metatibia long, narrow, widest at apex, ratio of width to length 1:3.9, sharply carinate dorsally, with three groups of spines, basal one at one-third, middle one at three-fifths, apical one at four-fifths of metatibial length, basally with a number of robust single setae; lateral face longitudinally convex, finely and moderately densely punctate, glabrous; apex shallowly, concavely truncate interiorly near tarsal articulation. Ventral edge finely serrate, with four robust equidistant setae. Tarsomeres finely punctate dorsally, with moderately dense, fine setae ventrally; metatarsomeres ventrally with a strongly serrate carina, subventrally with a second, smooth longitudinal carina; first metatarsomere a little shorter than following two tarsomeres combined, and little shorter than dorsal tibial spur. Protibia long, bidentate; anterior claws symmetrical, interior tarsal claws sharply pointed.

**Figure 1.**
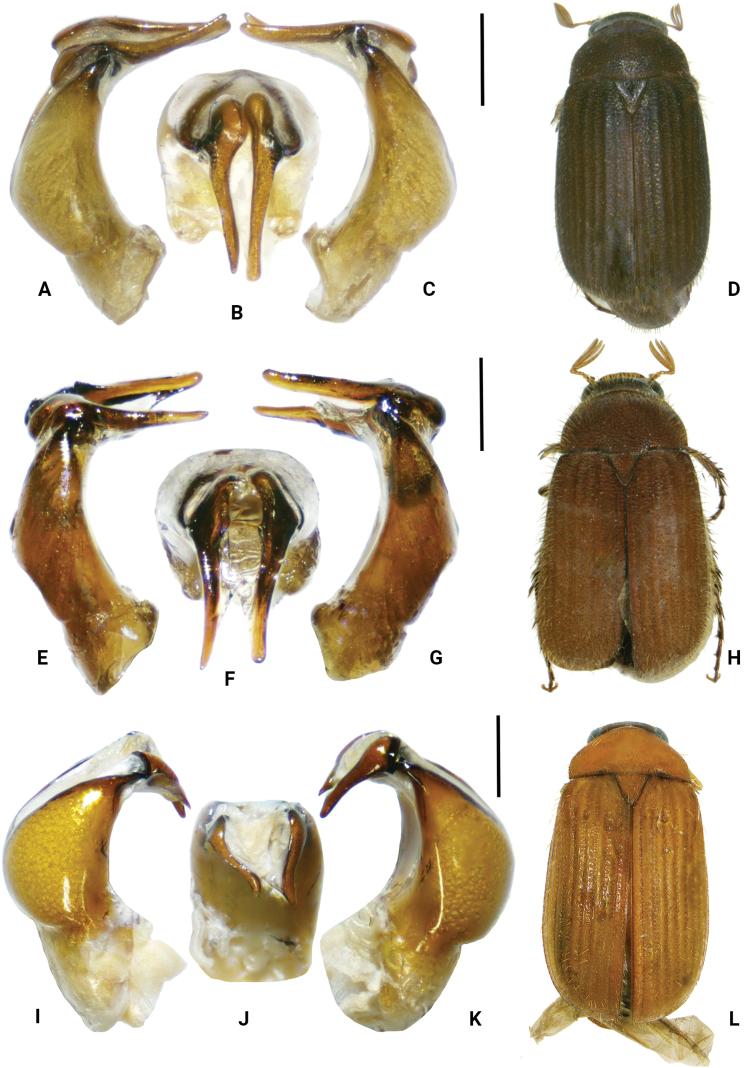
*Serica* species **A–D***S.breviantennalis* Liu, Ahrens, Li & Su, sp. nov. (holotype) **E–H***S.breviantennalis* (paratype) **I–L***S.allonanhua* Liu, Ahrens, Li & Su, sp. nov. (paratype). **A, E, I** aedeagus, left side view **C, G, K** aedeagus, right side view **B, F, J** aedeagus, dorsal view **D, H, L** habitus (not to scale). Scale bars: 0.5 mm (**A, B, C, E, F, G, I, J, K**).

***Aedeagus***: Fig. 1A–C, E–G. Habitus: Fig. 1D, H. Female unknown.

##### Diagnosis.

*Sericabreviantennalis* sp. nov. differs from *S.lupina* Arrow, 1946 and all other species of this species group in the short antenna and the shape of aedeagus; both parameres are long (subequal to half of the length of phallobase), nearly equal in length, parallel, and basally expanded to basal short lobes.

##### Etymology.

The name of the new species is derived from the combined Latin words *brevis* (short) and *antennalis* (antenna), with reference on the short antenna. (An adjective in nominative singular case.)

##### Variation.

Length 7.5–8.2 mm, elytral length 5.6–6.1 mm, width 4.0–4.3 mm. There is some variation of the parameres and lateral intervals of the elytra. Parameres of some specimens are closed, some are slightly open (Fig. 1A–C), and in others turned or rotated outside (Fig. 1E–G). Furthermore, the left paramere is sometimes curved externally (Fig. 1A–C) or sometimes dorsally (Fig. 1E–G). Lateral intervals of the elytra have a few moderately or distinctly dense, long, adpressed or erect setae.

#### ﻿ Serica allonanhua

Taxon classificationAnimaliaColeopteraScarabaeidae

Liu, Ahrens, Li & Su sp. nov.

B5F77989-B5D2-5BE3-AE91-6D187E818484

https://zoobank.org/976AA8D8-1FEE-4297-9F9F-194390DDCFCD

Figs 1I–L , 3

##### Type materials examined.

***Holotype***: ♂ [China] “Madeng Village, Jianchuan County, Dali, Yunnan Province, 31.V.2022, light trap, leg. Zhao Yuchen” (SENU). ***Paratypes***: 1 ♀ [China] “Black Tiger Mountain, Tianxin Township, Wuding County, Chuxiong, Yunnan Province, 20.V.2022, light trap, leg. Zhao Yuchen, Lu Jinbo” (SENU), 1 ♂ “LW-1065 / Yunnan, Lijiang, 2750 m Yulongxueshan, 2011-V-17. 27.013°N, 100.206°E” (ZFMK).

##### Description of the holotype.

Length 7.8 mm, elytral length 6.0 mm, width 4.2 mm. Body oblong, light reddish brown; frons reddish brown; some lateral and basal parts of elytra intervals dark reddish brown dull; legs yellowish brown; antenna yellow, with moderately long setae on dorsal surface.

Labroclypeus wide, trapezoidal; lateral margins moderately convex and convergent, producing a blunt angle with ocular canthus; anterior angles strongly convex; anterior margin distinctly emarginate medially; margins moderately reflexed; surface moderately convex and finely, densely punctate, with moderately long, erect setae; frontoclypeal suture distinct, moderately curved; ocular canthus moderately narrow (almost 1/3 of ocular width), smooth and glabrous, with one terminal seta. Frons with fine, moderately dense punctures, anteriorly and laterally with a few long setae. Antenna with nine antennomeres; club with three antennomeres, 1.8 times as long as remaining antennomeres combined, slightly reflexed. Eyes moderately large, ratio of diameter to interocular width 0.56. Mentum weakly elevated and flattened anteriorly.

***Pronotum*** moderately wide, widest at base; lateral margins moderately convex and convergent anteriorly; anterior angles acute, moderately produced, posterior angles blunt; anterior margin weakly convex, marginal line fine but complete; basal margin without marginal line; surface with coarse, dense punctures and minute setae, laterally bearing erect, long setae. Hypomeron not ventrally carinate. Scutellum triangular, with fine but very dense punctation and minute setae, with a triangular smooth area at middle.

***Elytra*** elongate, narrow, widest in posterior third; striae distinctly impressed, finely punctate; intervals weakly convex, finely and densely punctate, punctures concentrated along striae; glabrous, lateral intervals with a few moderately long, adpressed or erect setae; epipleural margin robust, ending at strongly rounded external apical angle of elytra; epipleura sparsely setose; apex of elytra with a very fine, membraneous rim of short microtrichomes.

***Ventral surface*** finely and densely punctate, glabrous. Metacoxa laterally with several longer setae. Mesosternum between mesocoxae almost as wide as mesofemur. Ratio of length of metepisternum to metacoxa 1:1.92. Abdominal sternites finely and moderately densely punctate, each with a row of coarse punctures; each puncture bearing a robust seta. Pygidium almost flat and finely, densely punctate, with moderately dense, long setae.

***Legs*** long, narrow, shiny; femora finely and sparsely punctate, with two longitudinal rows of setae, but anterior row reduced to a few setae. Metafemur with anterior margin acute, without adjacent serrate line; posterior ventral margin almost straight, weakly widened in apical half, ventrally and dorsally not serrate, sparsely setose. Metatibia long, narrow, widest at apex, ratio of width to length 1:3.5, moderately carinate dorsally, with three groups of spines, basal one at one-third, middle one at three-fifths, apical one at five-sixths of metatibial length, basally with a number of robust single setae; lateral face longitudinally convex, with fine, moderately dense punctae, glabrous; apex shallowly concavely truncate interiorly near tarsal articulation. Ventral edge finely serrate, with three robust, equidistant setae. Tarsomeres missing.

***Aedeagus***: Fig. 1I–K. Habitus: Fig. 1L.

##### Diagnosis.

*Sericaallonanhua* sp. nov. differs from *S.nanhua* and all other species of this species group in the light body colour and in the shape of aedeagus: the phallobase is strongly bent, the parameres are moderately long instead of only short (right and left paramere approximately one-third and one-fourth, respectively, as long as phallobase). Compared to *S.nanhua*, the parameres are distinctly longer, and in contrast to *S.baishuitaiensis* the parameres are straight in lateral view and narrow; the left paramere is slightly shorter than right one.

##### Etymology.

The name of the new species is derived from the combination of the Greek prefix *allo*- (different, other) and *nanhua*, in reference to the similarity of the new species to *Sericananhua*. (Used as a noun in apposition.)

##### Variation.

Female Length 7.6 mm, elytral length 5.8 mm, width 4.1 mm. Antennal club short, slightly shorter than remaining antennomeres combined; eyes smaller than in male, ratio of diameter to interocular width 1:0.40; pygidium weakly convex, ratio of metepisternum to metacoxa 1:1.57; ratio of width to length of metatarsomeres 1:4.1. Tarsomeres dorsally finely punctate, ventrally with moderately dense, fine setae; metatarsomeres ventrally with a distinct serrate carina, subventrally with a second smooth, longitudinal carina; first metatarsomere a little shorter than following two tarsomeres combined and almost as long as dorsal tibial spur. Protibia long, bidentate; anterior claws symmetrical; interior tarsal claws sharply pointed.

##### NCBI accession numbers.

A paratype of the species (voucher number LW-1065) was sequenced as *Serica* sp. by Liu et al. (2015b) for two mitochondrial (CO1: KJ959139, 16s: KJ959212) and one ribosomal marker (28S: KJ959057).

#### ﻿ Serica fengensis

Taxon classificationAnimaliaColeopteraScarabaeidae

Liu, Ahrens, Li & Su sp. nov.

708E3E78-591F-5245-B378-9D216F7E9B85

https://zoobank.org/49FA8835-21E3-4320-BFB0-7DD9BD547B99

Figs 2A–E , 3

##### Type materials examined.

***Holotype***: ♂ [China] “Feng County, Shaanxi, end of June.1974/ LW-174/ Asia Sericini 1325 spec.” (SENU). ***Paratypes***: 1 ♂ “LW-1034 China: Shaanxi Huangbaiyuan” (ZFMK), 1 ♂ “LW-1273 China: Shaanxi Liuba Huoshaodian, 2012-VI-19” (ZFMK), 1 ♂ “LW-1177 China: Shaanxi, Liuba, 2012-VI-22” (ZFMK), 1 ♂ “LW-1280 China: Shaanxi Liuba Huoshaodian, 2012.VI.23, 33.576°N, 106.961°E, 966m” (ZFMK), 1 ♂ “LW-1273bis China: Shaanxi Liuba Huoshaodian, 2012-VI-19” (ZFMK), 1 ♂ “X-DA3562 – China, Sichuan prov., W of Heishui, 2500m, 32.0465°N, 103.0168°E, 3.–10.VI.2012 leg. M. Murzin” (ZFMK).

##### Description of the holotype.

Length 8.4 mm, elytral length 6.0 mm, width 3.9 mm. Body oblong, narrow, dark brown; ventral surface, legs, and labroclypeus reddish brown; antenna yellow, with moderately dense, long setae on dorsal surface.

Labroclypeus wide, trapezoidal, shiny; lateral margins moderately convex and convergent, producing a blunt angle with ocular canthus; anterior angles moderately convex; anterior margin distinctly emarginate medially; margins moderately reflexed; surface flat, finely and densely punctate, with numerous long and erect setae; frontoclypeal suture distinct, moderately curved; ocular canthus long and narrow (ca 1/3 of ocular width), smooth and glabrous, with two terminal setae. Frons dull, with fine, dense punctures and dense, long, erect setae. Antenna with nine antennomeres; club with three antennomeres, 2.5 times as long as remaining antennomeres combined, strongly reflexed. Eyes moderately large, ratio of diameter to interocular width 0.67. Mentum weakly elevated and anteriorly flattened.

***Pronotum*** moderately wide, widest at middle; lateral margins moderately convex and convergent anteriorly, slightly narrowed towards base; anterior angles rectangular, moderately produced, posterior angles blunt; anterior margin weakly convex, marginal line fine and complete; basal margin without marginal line; surface with fine, dense punctures and minute setae in punctures, with sparse, erect, long setae. Hypomeron not ventrally carinate. Scutellum triangular, with fine, dense punctation and minute setae; basal midline with a triangular smooth area.

***Elytra*** elongate, narrow, widest in posterior third; striae distinctly impressed, finely punctate; intervals weakly convex and finely, densely punctate; glabrous, lateral intervals with a few moderately long, erect setae, otherwise with only minute setae in punctures; epipleural margin robust, ending at convex external apical angle of elytra; epipleura sparsely setose; apex of elytra with a fine, membraneous rim of short microtrichomes.

***Ventral surface*** finely, densely punctate, and metasternum sparsely setose. Metacoxa glabrous, laterally with numerous, robust, fine, longer setae. Mesosternum between mesocoxae almost as wide as mesofemur. Ratio of length of metepisternum to metacoxa 1:1.52. Abdominal sternites finely, moderately, and densely punctate, each sternite with a row of coarse punctures, each puncture bearing a robust seta. Pygidium almost flat and finely, densely punctate, with moderately dense, short and long setae.

***Legs*** long, narrow, shiny; femora finely, sparsely punctate, with two longitudinal rows of setae, but anterior row reduced to a few setae. Hind legs missing in holotype. Tarsomeres dorsally finely punctate, ventrally with moderately dense, fine setae. Protibia long, bidentate; anterior claws symmetrical; interior tarsal claws sharply pointed.

**Figure 2.**
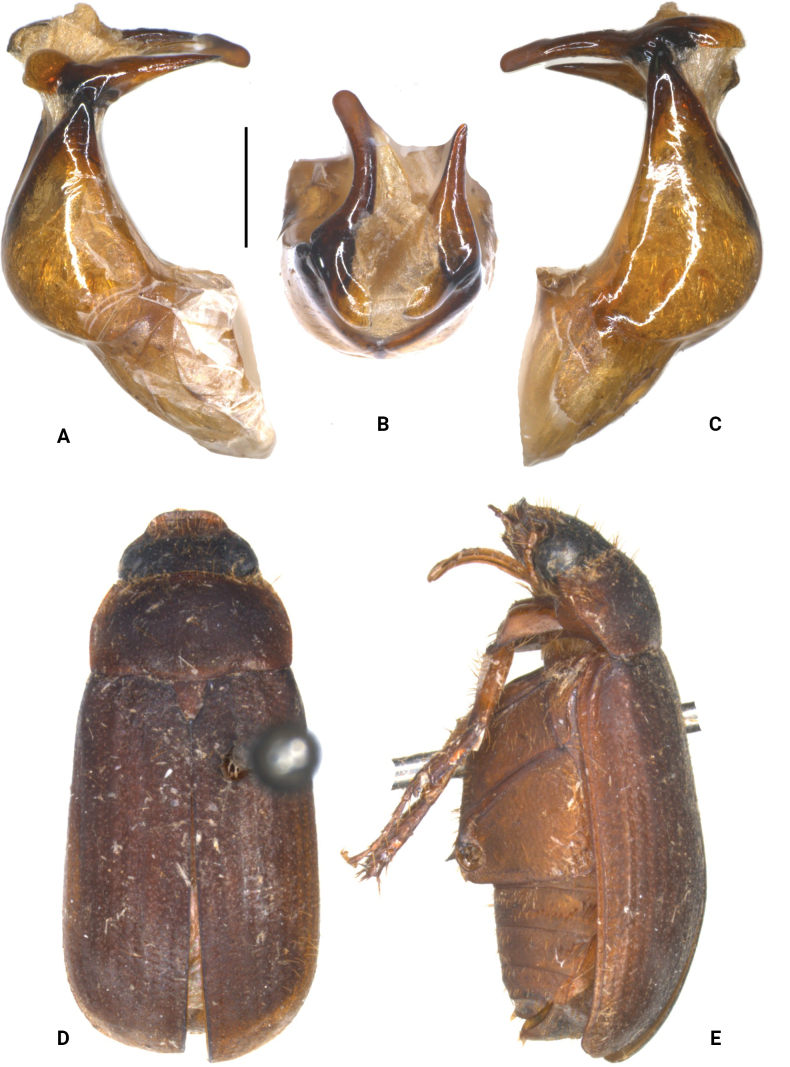
**A–E***S.fengensis* Liu, Ahrens, Li & Su, sp. nov. (holotype) **A** aedeagus, left side view **C** aedeagus, right side view **B** aedeagus, dorsal view **D** habitus, dorsal view **E** habitus, lateral view. Scale bar: 0.5 mm (**A–C**).

***Aedeagus***: Fig. 2A–C. Habitus: Fig. 2D. Female unknown.

##### Diagnosis.

*Sericafengensis* sp. nov. differs from the quite similar *S.allonanhua* sp. nov. in the long basal lobes of the parameres; these lobes are absent in the latter species.

**Figure 3.**
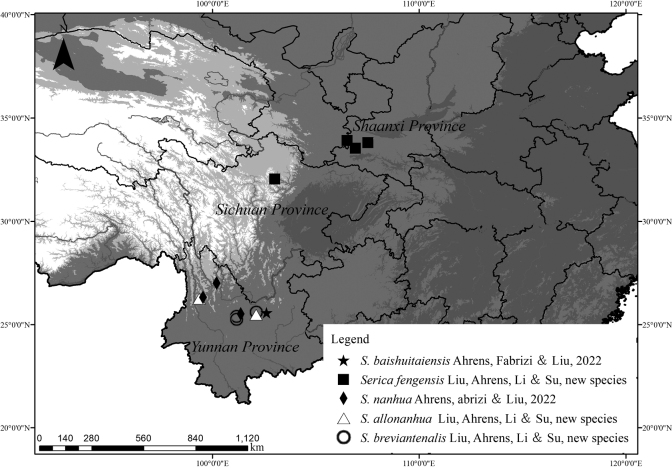
The distribution map of *Sericabreviantennalis* Liu, Ahrens, Li & Su, sp. nov., *S.allonanhua* Liu, Ahrens, Li & Su, sp. nov., *S.fengensis* Liu, Ahrens, Li & Su, sp. nov., including updated records of *S.nanhua* Ahrens, Fabrizi & Liu, 2022 and *S.baishuitaiensis* Ahrens, Fabrizi & Liu, 2022.

##### Etymology.

The name of the new species is derived from the location of type locality in Feng County. (Used as an adjective in nominative singular case.)

##### Variation.

Length 6.8–8.4 mm, elytral length 5.3–6.5 mm, width 3.8–4.0 mm. Most paratypes with dense pilosity on pronotum and elytra, suggesting that pilosity of holotype has been widely abraded. Metafemur with anterior margin acute, without adjacent serrate line; posterior ventral margin almost straight, weakly widened in apical half, ventrally and dorsally not serrate, sparsely setose. Metatibia long and moderately narrow, widest at middle, ratio of width to length 1:3.3, dorsally sharply carinate, with two groups of spines, basal one shortly behind middle, apical one at four-fifths of metatibial length, basally with a number of robust, single setae in line; lateral face longitudinally convex, coarsely and densely punctate, with a numerous short setae in basal half; apex shallowly concavely truncate interiorly near tarsal articulation. Ventral margin finely serrate, with five or six robust equidistant setae. Metatarsomeres ventrally with a strongly serrate carina, subventrally with a second, smooth, longitudinal carina; first metatarsomere distinctly shorter than following two tarsomeres combined and little longer than dorsal tibial spur.

##### NCBI accession numbers.

Paratypes of the species have been sequenced as *Serica* sp. by Liu et al. (2015b) for two mitochondrial and one ribosomal marker (voucher number LW-1034: 16s: KJ959207, CO1: KJ959135, 28s: KJ959052; LW-1273: 16s: KJ959208, CO1: KJ959136, 28s: KJ959053). The locality data of the LW-1273 specimen were erroneously given in the supplementary table 1 of Liu et al. (2015b) as “China: Yunnan, Pianma” instead of “Shaanxi Liuba Huoshaodian, 2012-VI-19”.

### ﻿New records

#### ﻿ Serica nanhua

Taxon classificationAnimaliaColeopteraScarabaeidae

Ahrens, Fabrizi & Liu, 2022

634DCC80-426B-524B-B7AE-747B01161B20

##### Additional materials examined.

1 ♂ [China] “Qianchang town, Yao ‘an, Chuxiong, Yunnan Province, 23.V.2022, light trap, 2095 m, 25.52360(N), 101.37449(E), leg. Zhao Yuchen” (SENU), 1 ♂ [China] “Dali Jianchuan, Yunnan Province, 1.VI.2022, light trap, 2200m, 26.321291°N, 99.541980°E, leg. Zhao Yuchen” (SENU).

#### ﻿ Serica baishuitaiensis

Taxon classificationAnimaliaColeopteraScarabaeidae

Ahrens, Fabrizi & Liu, 2022

6C421111-C34A-50D2-935A-39F2C615D372

##### Additional materials examined.

3 ♂♂ [China] “Black Tiger Mountain, Tianxin Township, Wuding County, Chuxiong, Yunnan Province, 20.V.2022, light trap, 1160 m, 25.49412°N, 102.113091°E, leg. Zhao Yuchen, Lu Jinbo” (SENU).

## Supplementary Material

XML Treatment for Serica breviantennalis

XML Treatment for Serica allonanhua

XML Treatment for Serica fengensis

XML Treatment for Serica nanhua

XML Treatment for Serica baishuitaiensis

## ﻿Additional information

### Author ORCIDs

Xie Su https://orcid.org/0009-0004-1723-2542

Wei Li https://orcid.org/0009-0001-0195-7889

Wan-Gang Liu https://orcid.org/0000-0003-4788-7967

Dirk Ahrens https://orcid.org/0000-0003-3524-7153

Yang Liu https://orcid.org/0000-0003-0301-0186

### Data availability

All of the data that support the findings of this study are available in the main text.
